# An Origami-Based Soft Robotic Actuator for Upper Gastrointestinal Endoscopic Applications

**DOI:** 10.3389/frobt.2021.664720

**Published:** 2021-05-10

**Authors:** Manish Chauhan, James H. Chandler, Animesh Jha, Venkataraman Subramaniam, Keith L. Obstein, Pietro Valdastri

**Affiliations:** ^1^ Science and Technology of Robots in Medicine (STORM) Laboratory, School of Electronics and Electrical Engineering, University of Leeds, Leeds, United Kingdom; ^2^ School of Chemical and Process Engineering, University of Leeds, Leeds, United Kingdom; ^3^ Faculty of Medicine and Health, University of Leeds, Leeds, United Kingdom; ^4^ Division of Gastroenterology, Hepatology and Nutrition, Vanderbilt University Medical Center, Nashville, TN, United States

**Keywords:** soft robotics, upper G.I. endoscopy, volume and pressure characterization, monolithic soft actuators, soft actuator model, soft actuator fabrication

## Abstract

Soft pneumatic actuators have been explored for endoscopic applications, but challenges in fabricating complex geometry with desirable dimensions and compliance remain. The addition of an endoscopic camera or tool channel is generally not possible without significant change in the diameter of the actuator. Radial expansion and ballooning of actuator walls during bending is undesirable for endoscopic applications. The inclusion of strain limiting methods like, wound fibre, mesh, or multi-material molding have been explored, but the integration of these design approaches with endoscopic requirements drastically increases fabrication complexity, precluding reliable translation into functional endoscopes. For the first time in soft robotics, we present a multi-channel, single material elastomeric actuator with a fully corrugated design (inspired by origami); offering specific functionality for endoscopic applications. The features introduced in this design include i) fabrication of multi-channel monolithic structure of 8.5 mm diameter, ii) incorporation of the benefits of corrugated design in a single material (i.e., limited radial expansion and improved bending efficiency), iii) design scalability (length and diameter), and iv) incorporation of a central hollow channel for the inclusion of an endoscopic camera. Two variants of the actuator are fabricated which have different corrugated or origami length, i.e., 30 mm and 40 mm respectively). Each of the three actuator channels is evaluated under varying volumetric (0.5 mls^-1^ and 1.5 mls^-1^ feed rate) and pressurized control to achieve a similar bending profile with the maximum bending angle of 150°. With the intended use for single use upper gastrointestinal endoscopic application, it is desirable to have linear relationships between actuation and angular position in soft pneumatic actuators with high bending response at low pressures; this is where the origami actuator offers contribution. The soft pneumatic actuator has been demonstrated to achieve a maximum bending angle of 200° when integrated with manually driven endoscope. The simple 3-step fabrication technique produces a complex origami pattern in a soft robotic structure, which promotes low pressure bending through the opening of the corrugation while retaining a small diameter and a central lumen, required for successful endoscope integration.

## Introduction

Nature has been a prime source of inspiration for the development of a broad family of continuum robots (CR) for use in medical applications (Burgner-Kahrs et al., 2015; da Veiga et al., 2020). Invertebrate animals like the octopus and muscular hydrostats such as the flexible trunk of the elephant have inspired the development of these compliant structures (Cianchetti et al., 2014; Renda et al., 2014; Chen et al., 2020) which inherently conform to the shape and structure of their surroundings. The compliance of CR structures comes from the employment of a continuous underlying elastic backbone, which articulates to form smooth curves under actuation. Actuation of continuum robots depends on the targeted application. The various methods of CR actuation include tendon cables (York et al., 2015; Chauhan et al., 2019), concentric tubes (Rucker et al., 2010; Bedell et al., 2011), shape memory alloys (Blanc et al., 2017), hydraulic fluid (Campisano et al., 2017), pneumatic pressure (Garbin et al., 2018a), and magnetics (Lloyd et al., 2020) etc. Of these, a particularly prevalent CR class is that of pneumatically driven actuators with hyper-elastic-material-based bodies. Soft robotic actuators of this type have been explored for many use-cases (Elsayed et al., 2014; Drotman et al., 2018; Yin et al., 2019), however, they have proven particularly well suited to endoscopic applications (Martinez et al., 2013; Garbin et al., 2018b; Gorissen et al., 2018; Gifari et al., 2019; Lenssen, 2019; Lenssen et al., 2019; Naghibi et al., 2019) etc.

An important potential application for pneumatically driven flexible endoscopes is in upper gastrointestinal (UGI) screening (inspection of the esophagus, stomach, and duodenum); mainly due to the potential benefits of inflicting less-pain and discomfort, as well as being associated with lower-costs compared to commercial gastroscopes which require sedation and additional support; creating a burden of care for both the hospital and the patient’s family. Gastroscopes form a critical tool for detection of pathologies such as cancer. The design of a gastroscope specifically is a function of biological constraints, which dictate its key features, like i) a diameter between 4.9 to 12.8 mm (Blinman, 2010; Marlicz et al., 2020) to allow insertion through the nose or mouth, ii) an optimum balance of compliance and stiffness for entering through the vocal folds, iii) navigation capability through and within the esophagus, stomach and duodenum, iv) maintaining dynamic stability with minimal vibrational disturbance, v) adequate visualization while maintaining minimal dimensions, and vi) safety for the patient and clinician. Pneumatically driven soft robotic actuators are desirable as they have the potential to satisfy these requirements. However, their bending performance is dependent on the choice of design features, i.e., i) number of inflatable channels, ii) length and diameter, iii) choice of elastomeric material for desired flexibility or stiffness, iv) provision of an endoscopic camera or other features like a biopsy, suction, or flushing/cleaning channel etc., and v) fabrication method. A balanced selection across these features is necessary to achieve suitable stiffness, stability, and localization control of the endoscopic tip.

The choice of design features of pneumatic actuators is a function of desired degrees of freedom and the cross-sectional area of the inflatable channel. Both features influence the actuator diameter. Pneumatic actuators with single channel (Gorissen et al., 2018) and four channels (Gifari, 2018; Naghibi et al., 2019) have been designed, but it is established that three inflatable channels (configured at angular separation of 120°) are sufficient to reach positions covering a three-dimensional workspace (Lenssen, 2019). When air pressurizes the channels of a soft robotic actuator, resultant elongation and thus bending occur. In unrestricted designs, radial bulging or expansion will occur simultaneously, resulting (temporarily) in an increase in overall actuator diameter. Some methods of constraining the radial expansion and promoting more efficient bending include the addition of strain limiting fiber, mesh or sheet layers (Krishnan et al., 2013; Bishop-Moser and Kota, 2015; Polygerinos et al., 2015), optimization of chamber design in monolithic actuators (Elsayed et al., 2014), and the use of multiple materials with dissimilar stiffness properties (Martinez et al., 2013; Mosadegh et al., 2014b; Mosadegh et al., 2014a; Sun et al., 2017). Another method of controlling the radial expansion is to introduce a corrugated pattern along the cross-section of the channel (Ilievski et al., 2011; Mosadegh et al., 2014b; Mosadegh et al., 2014a; Wang et al., 2018). A corrugated pattern allows greater strain because it lowers the internal volume of the hollow channel, induces directional asymmetry, achieves a higher bending angle and overcomes the limitation of slow actuation (Mosadegh et al., 2014b; Mosadegh et al., 2014a). Furthermore, it also promotes constant curvature bending with a minimal radius of curvature for precise and repeatable control over the endoscopic tip (Gorissen et al., 2017). Studies report that an increase in the bending angle is possible with the division of the actuator length into smaller zones or balloons (Morimoto et al., 2011). However, the inclusion of multiple corrugated channels into a small diameter actuator, suitable as an endoscope, remains challenging.

In a previous study from the authors, we introduced the development of a mechanical continuum joystick for driving three individual corrugated bellows (configured at 120° angle around the central axis) (Garbin et al., 2018b). Later, we also introduced the possibility to generate an internal corrugated pattern with a smooth external design through the use of parallel helical cores (Chandler et al., 2020). The parallel helical actuator reported the packing of three inflatable helical channels in an overall diameter of 9.54 mm. This delivers high packing efficiency, as three complex structured channels were accommodated within the minimal possible cross-sectional area or diameter (i.e. number of channels/actuator diameter). Though this design approach is well suited for many soft robotic applications, incorporation of a hollow channel along its length is not readily achieved without significantly increasing the overall diameter; thus limiting the design’s utility in endoscopic applications.

In this paper we present, for the first time, an origami-inspired corrugated 3-channel monolithic soft robotic design. The presented Origami actuator (OA) offers specific functionality for upper gastrointestinal endoscopic applications. The features introduced in this design include, i) production of multi-channel monolithic structure at 8.5 mm diameter, ii) incorporation of the benefits of corrugated design in single material (i.e., limited radial expansion, improved bending efficiency and high packing efficiency), iii) design scalability (length and diameter), and iv) incorporation of a central hollow channel for the inclusion of an endoscopic camera or tool. The following sections introduce the Origami concept and method for incorporating features into actuator designs, fabrication technique, experimental evaluation of 3D workspace due to variation of volume and pressure of air, and changes in reachability due to repeated pressurized inflation. Stability and achievable workspace assessment (by repeated pressurization) of the actuator is done after integration with the endoscope handle (Garbin et al., 2018b).

## Principle of Design

Various studies have taken inspiration from the Japanese art of folding paper (i.e., Origami) to create, for example, three-dimensional metamaterial (Overvelde et al., 2016), artificial muscles (Li et al., 2017), spring-inspired programmable robots (Hu et al., 2020), and forceps (Edmondson et al., 2013). Origami induces inherent flexibility in rigid structures by adding multiple folds and creases. These fold lines behave like hinges or joints in a continuum structure, which can be constricted and promote motion in specific directions.

**FIGURE 1:**
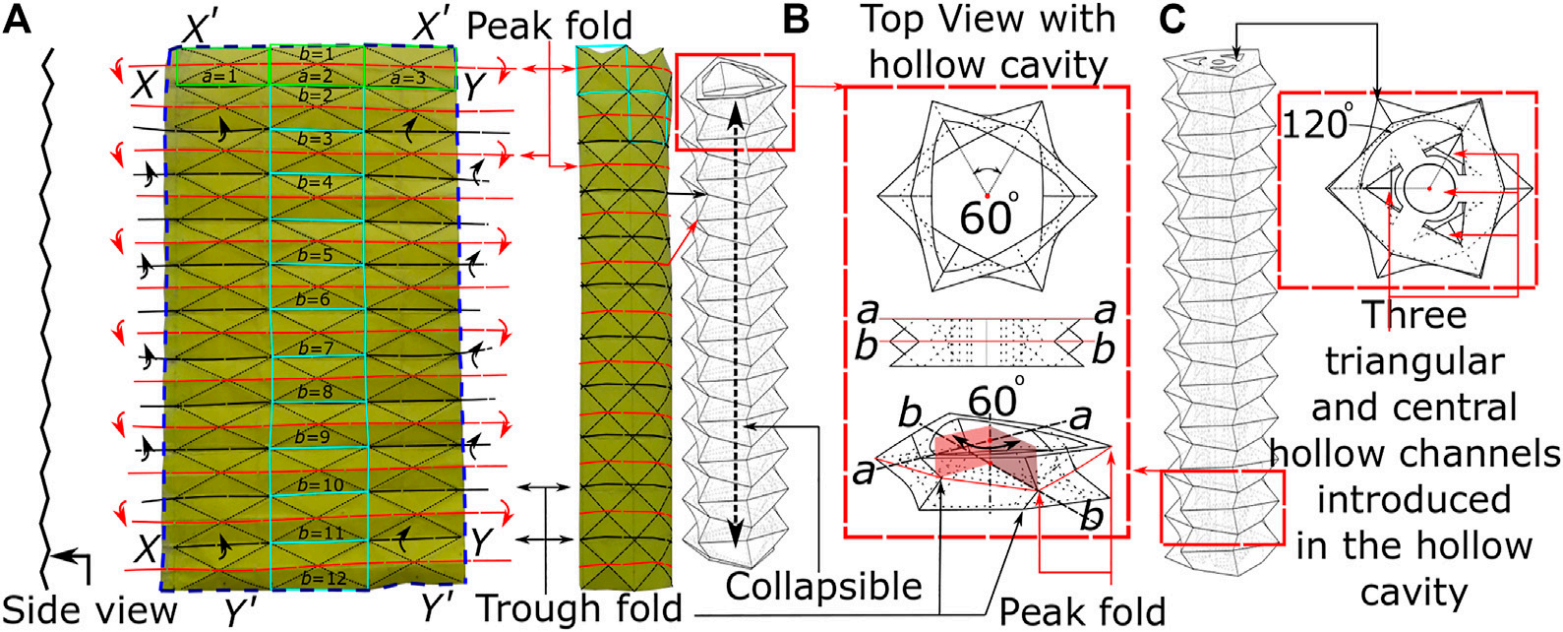
The principle of origami design **(A)** the origami pattern on paper **(B)** building block of PFP **(C)** actuation channel and central lumen.

With the intended use for endoscopic applications, one of the key design requirements for flexible endoscopes is the ability to have controlled bending of the soft robotic tip with pneumatic pressure. In specific configurations, Origami can offer functionality of greater strain for low-pressure actuation, high packing efficiency, and minimum strain energy when folded in lightweight structures. Figure 1 shows the paper model of the origami pattern utilized in this presented method, known as the ‘Pineapple Folding Pattern’ (PFP). This pattern is a combination of repeated adjacent peak (or hill) and trough (or valley) folds in a single row (*a* = 1, 2, 3; highlighted by green rectangle). This pattern in a single row repeats along the length of the paper to create the PFP (*b* = 1 to 12) (refer Figure 1A). This zigzag-corrugated pattern allows it to remain flexible along the length and breadth of the paper. Once the fold creases are made, the opposite edges are joined together to form a tubular structure. Depending upon the choice of edges, like joining X−X and Y−Y or $X^{'}-X^{'}$ and $Y^{'}-Y^{'}$, the pattern can fold and expand longitudinally or radially respectively. A radially folding PFP pattern was used to develop a novel foldable stent graft (Kuribayashi, 2004; Ma, 2011). Similarly, a soft pneumatic gripper with a tendon driven soft origami pump was built using a Kresling pattern (Kim et al., 2020), which is similar to PFP. Another pattern similar to PFP, i.e., stacked octagonal layers by Mihoko Tachibana was used to create an under actuated robotic gripper; TWISTER Hand (Lee et al., 2020). Martinez et al (2012) reported programmable paper-elastomeric composites origami, where a paper-based bellows origami pattern (similar to PFP) was molded with elastomeric material to prototype an elongating actuator. A yoshimura origami pattern (Paez et al., 2016) was explored to produce a soft pneumatic actuator. The same pattern was utilized (Santoso and Onal, 2020) to fabricate a PET sheet into a foldable origami structure for producing continuum robotic arm. A yoshimura pattern was also used to create an optical sensing actuator for underwater manipulation (Shen et al., 2020). Various applications with origami patterns have been explored but for the first time in the field of tip articulation of flexible endoscopes, we present a longitudinally folding tubular structure by joining X-X and Y-Y edges. The top view (Figure 1B) of this tubular structure resembles a hollow hexagon, which is formed by two layers of triangular shape (a and b**)** offset by 60^o^ angle. This angular offset allows bending flexibility and easy change of the bending plane along the length of the tubular structure. An additional benefit of this feature allows the tubular structure to collapse and elongate, or allow easy change in length. Incorporation of these structural benefits in soft robotic pneumatic structure is possible by introduction of three triangular channels in the hollow cavity (Figure 1C). The inherent central hollow channel allows access from the proximal to distal end, e.g. to pass an endoscopic camera. The uniqueness of this design allows it to remain stiff and stable as a self-supporting structure while having the capability (due to its longitudinal folding feature) to articulate flexibly and change bending planes with ease and stability. The following section explains the fabrication method for obtaining soft robotic OA.

## Fabrication Method

**FIGURE 2:**
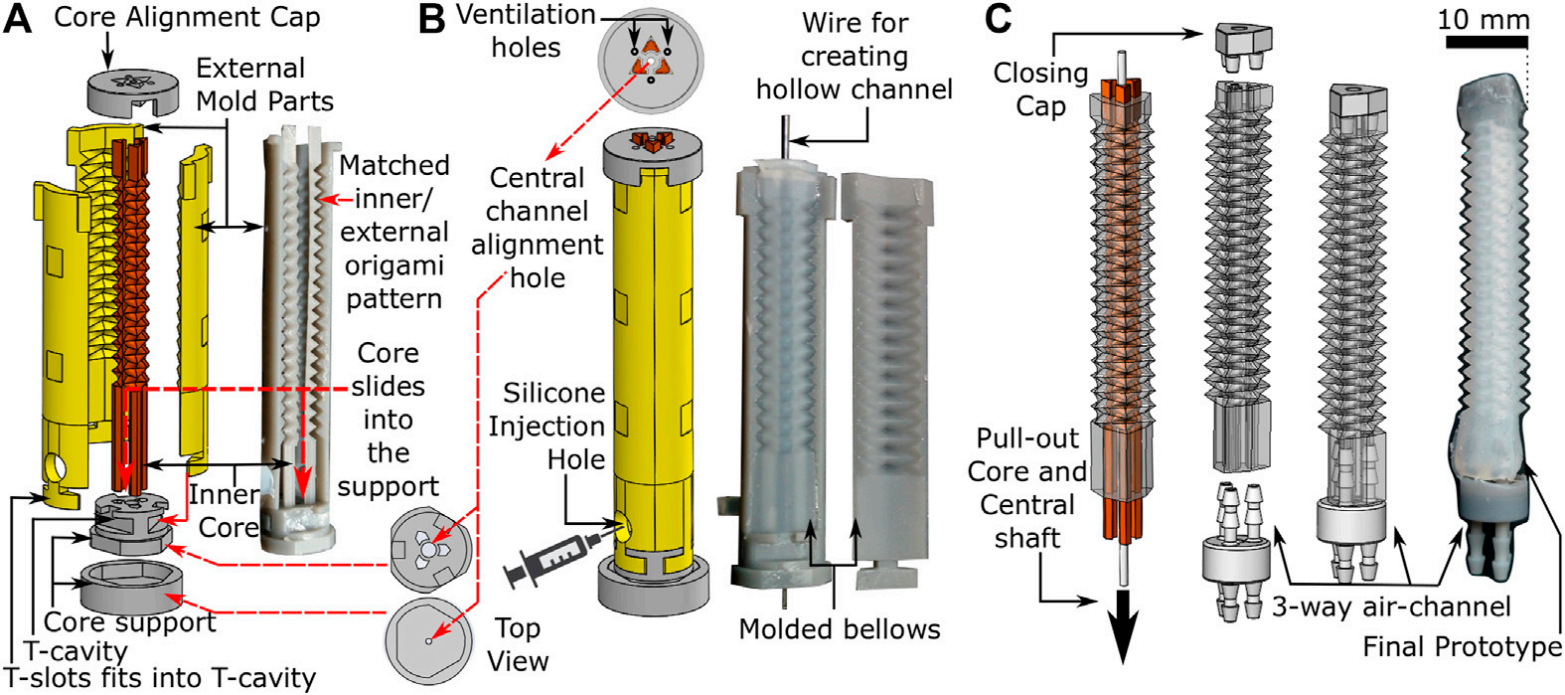
Fabrication process of origami actuator **(A)** assembly of three piece internal cores and external mold parts **(B)** assembled mold parts with silicone injection hole **(C)** pulling out-off the inner core and central shaft to form final prototype with three-way air-channel.

Fabrication of the OA (Figure 2) involves a 3-step molding process, i) assembly of mold parts, ii) injection of elastomeric material into the mold, and iii) demolding and final preparation of the cured OA. Figure 2A shows the 3D printed design of the customized mold, which is comprised of three internal cores within a six-piece external mold. The surface design of both the cores and the external mold parts replicate the peak and trough folds of the PFP (Figure 1). The three-piece inner core slides vertically into a specially designed two-piece core support. The external mold also fits vertically into the core-support through T-slots and covers the inner core circumferentially. Both internal core and external mold are assembled on the core support such that the PFP features precisely match (Figure 2A) while maintaining a gap for molding purposes. The two-piece core support also includes a hole along its central axis for supporting a wire vertically (φ 1 mm) along the length of the assembled mold until the core alignment cap, which covers the top end of the assembled mold and allows the wire to pass through its central hole. The core alignment cap also contains triangular slots for aligning the three-piece inner core and avoiding any molding defects.

To form OAs, silicone (Dragon Skin 10, Smooth-ON, United States) was injected into the assembled mold (refer to Figure 2B) after mixing and degassing it in a vacuum mixer (ARV-310, Thinky, United States). The silicone material was injected gradually into the mold, such that any air could escape through the ventilation holes in the core alignment cap. Once the mold was filled completely, it was cured at 60^o^C for 60 min. Figure 2B shows the cured origami structure, which was demolded by removing the external mold parts carefully to obtain the OA with inner cores (refer to Figure 2C). Thereafter, syringes were used to flush air through both ends of the molded structure to break any adhesions of silicone to the inner core surface. The scale of features on the corrugated inner core are small enough, i.e., a shallow triangular repeating pattern, that once the adhesions with the mold wall are broken it is easy to pull out the inner core without damaging the actuator walls to obtain the three-channeled OA. The central wire was also removed using this method to obtain a hollow central channel. The top and the bottom ends of the actuator were sealed with a closing cap and a three-way air-channel respectively using silicone adhesive (Sil-Poxy, Smooth-ON, United States). Connection of the pressure source to the three-way air channel facilitated actuation.

## Design Parameters and Mathematical Model

**FIGURE 3:**
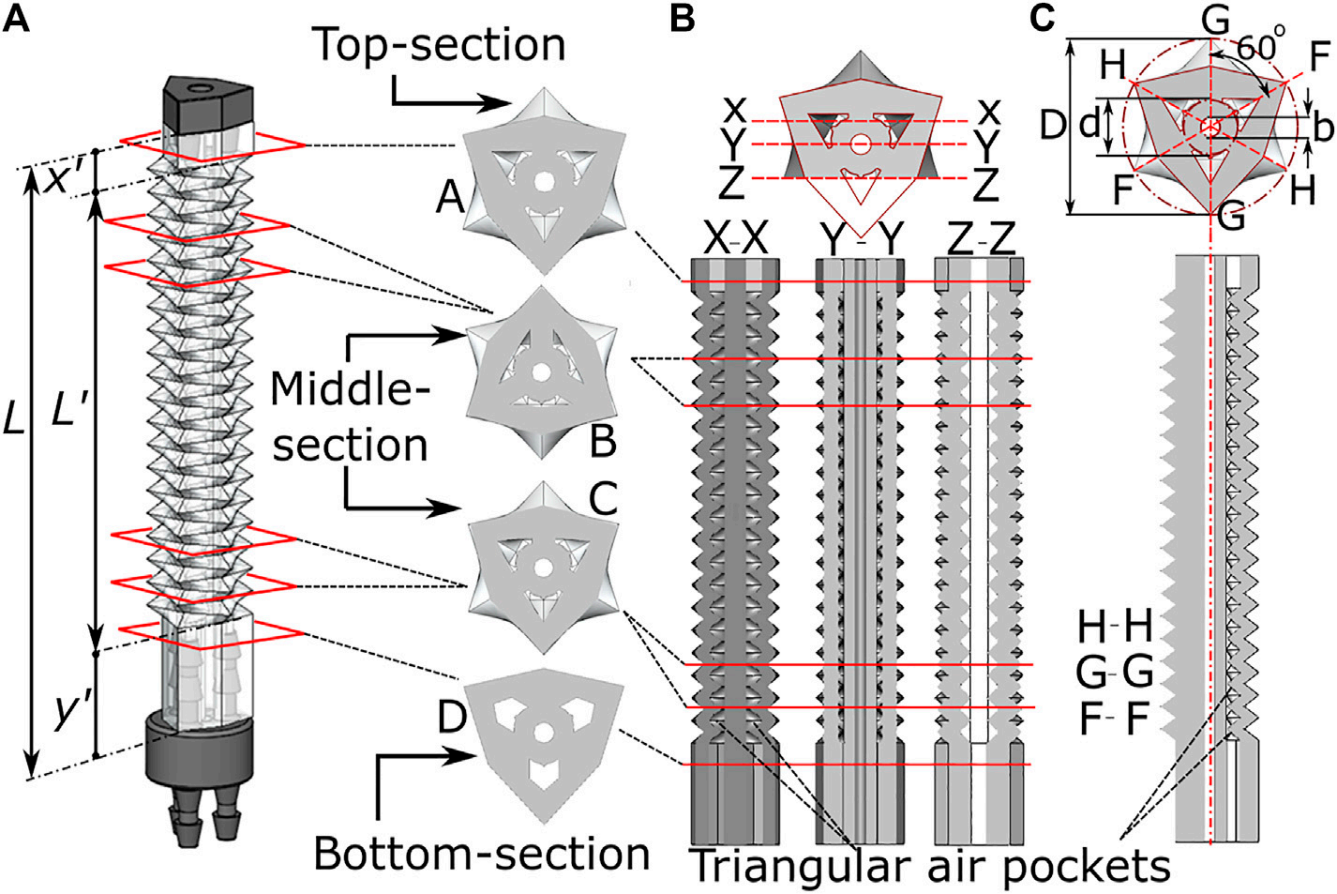
Internal features of the origami monolithic actuator, showing **(A)** the completed actuator with three-channels and axial cross-section; longitudinal cross-section at **(B)** X-X, Y-Y and Z-Z plane and **(C)** H-H, G-G and F-F plane along with dimensional parameters.

The above presented fabrication technique produces a complex feature set in an elastomeric material, whose design parameters are shown in Figure 3. The total length L of the OA is comprised of three sections of lengths $x^{'}$, $L^{'}$ and $y^{'}$. The sections with length $x^{'}$ (2.90 mm) and $y^{'}$ (9 mm) are designed to accommodate the closing cap and three-way air-channel respectively (refer to Figure 2C). The cross-sectional views of these two parts of OA are shown by sectional-plane A and D. The corrugated PFP is designed in the middle of the OA with length $L^{'}$. Sectional planes B and C show the triangular cross-section layers, which are arranged along the length of the OA, alternating with an angular offset of 60° (Figure 1B). These cross-sectional views (A, B, C, and D) are matched with the longitudinal cross-sections at X-X, Y-Y, and Z-Z plane in Figure 3B. The corrugated PFP pattern is observed to create small air pockets of triangular shape along the actuator length. The true shape of the inflatable channel can be seen through any one of the cross-sectional planes H-H, G-G, and F-F. These planes can be seen in Figure 3C, at an angular gap of 60°.

The length $L^{'}$ represents the active region of the OA, containing corrugated air channels that produce elongation upon the application of pneumatic pressure. This elongation results from two factors: i) the opening of the corrugations (i.e., increase in corrugation angle), and ii) the elastic strain in the material of the outer wall of the channel. It is proposed that the OA design offers the benefit of corrugation opening i) allowing significant elongation of each pressurized channel before appreciable strain is induced into the elastomeric material of the OA wall ii). This behavior would promote higher bending angles at lower pressure with a more linearized response within this bending range; which may be tuned through parameter selection to fall within the desired operating range of bending angles (θ).

**FIGURE 4:**
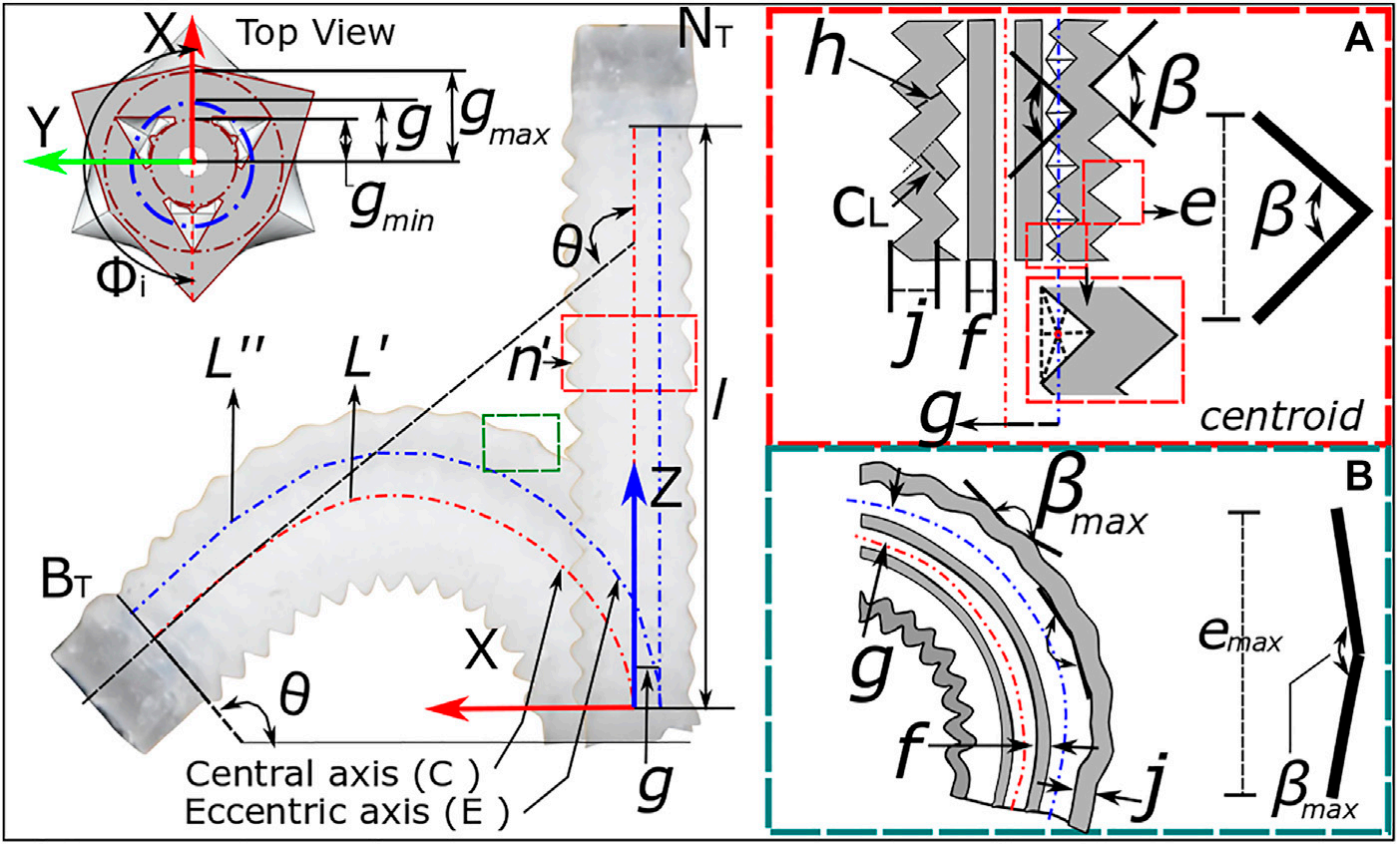
Bending kinematics for OA showing the design parameters for single channel actuation from **(A)** neutral position to **(B)** bending of actuator.

To predict the influence of design parameters and corrugation behavior on the maximum bending angle expected due to corrugation opening alone, it is possible to model the OA through the use of the constant curvature assumption (Webster III and Jones, 2010). Considering first the general case of a three-channeled pneumatic actuator with a channel configuration as presented in Figure 4 under actuation of a single channel, it is possible to relate the length of the central ‘backbone’ l to the effective length of the pressurized channel $L^{''}$ through the bending angle θ and radial distance g between the central ‘backbone’ and channel wall, as

$$l=L^{''}-\theta g$$ (1)

This assumes an angle of the actuated chamber relative to the *x*-axis (refer Figure 4), $\phi_{i}$ of 180^o^. Furthermore, the effective central length l may be determined as

$$l=\left(L^{''}+2L_{aux}\right)/3$$ (2)

where $L_{aux}$ represents the lengths of inactive channels; considered to remain equal to $L^{'}$ during actuation. Combining Eqs. 1, 2 gives an expression for bending angle θ as a function of the channel lengths and their radial positioning in the OA, as

$$\theta =\frac{\;L^{''}-\left(\left(L^{''}+2L_{aux}\right)/3\right)}{g}$$ (3)

The generic form of Eq. 3 can be expanded to include the OA design parameters. Specifically, by considering the geometric properties of the corrugations, the maximum bending angle under the corrugation opening may be predicted. As mentioned above, this region of operation may provide desirable characteristics of lower pressured bending and a more linear response (due to operating in the low strain region of the hyperelastic material properties). The estimated channel length as a result of corrugation opening is a function of the number of corrugations ($n^{'})$, the corrugation wall length ($c_{L})$ and the maximum corrugation opening angle ($\beta_{max}$). With reference to Figure 4, it is apparent that these parameters may be related to give the maximum length per corrugation ($e_{max})$, as

$$e_{max}=2c_{L}\text{sin}\left(\beta_{\max}/2\right)$$ (4)

and Eq. 4 will relate to the overall channel length $L^{''}$ under maximum opening as

$$L^{''}=n^{'}e_{max}$$ (5)

Equation 5 can thus be utilized with Eq. 3 to predict a maximum bending angle from corrugation opening $\theta_{max}$ for the given design parameters.

## Design Implementation

**FIGURE 5:**
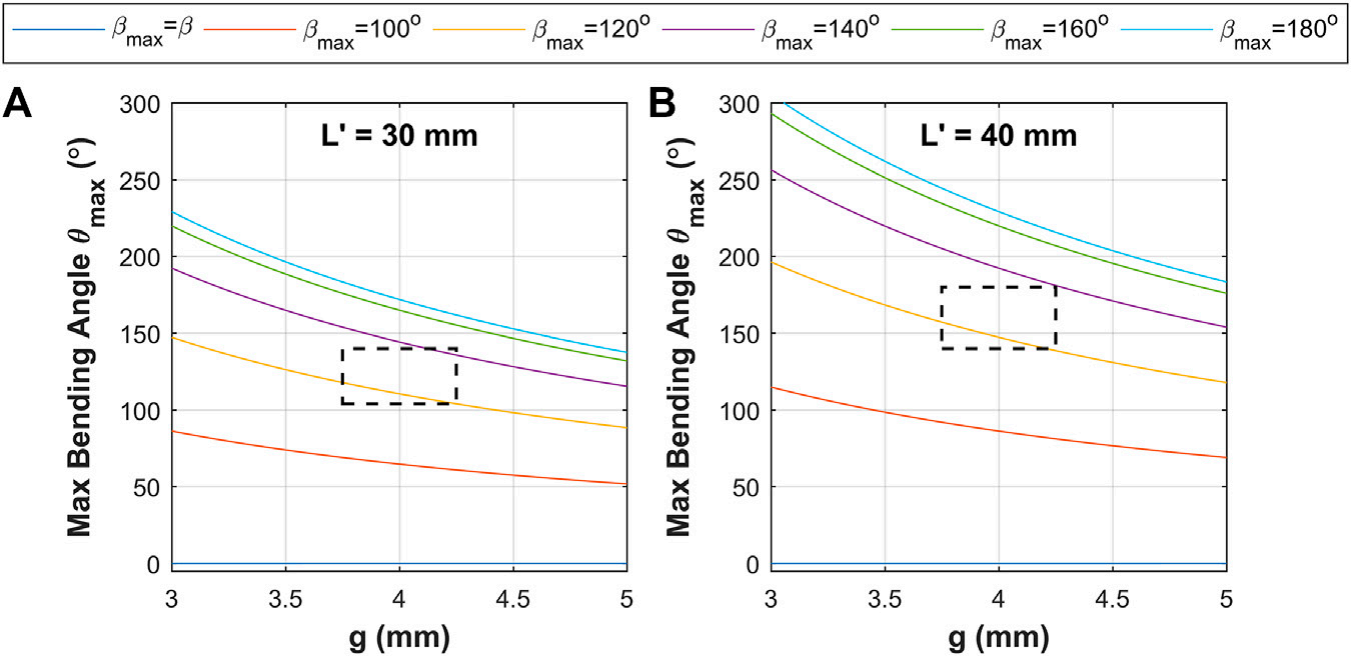
Model prediction results of maximum bending angle under corrugation opening $\theta_{max}$ for variation in chamber radial distance g and maximum corrugation opening angle $\beta_{max}$ for **(A)** $L^{'}$ = 30 mm and **(B)** $L^{'}$ = 40 mm; black dashed line boxes indicate estimated operating window for proposed designs.

With consideration of the above model, a design space was constructed based on the parameters presented in Table 1. These primarily consider two length cases constructed of 15 and 20 corrugations to deliver corrugated sections lengths $L^{'}$ of 30 mm and 40 mm respectively (total respective lengths of the complete OAs being 41.90 mm and 51.90 mm). As the effective channel actuation radius g and maximum corrugation opening angle $\beta_{max}$ are not possible to define exactly, the predicted $\theta_{max}$ over a range of these parameters was considered for the two length cases; as shown in Figure 5. Based on the geometry of the OA, the radial distance g should fall between the inner and outer wall of the actuated channel, with a tendency toward a higher value as pressure increases due to any small amounts of radial expansion. Thus, the estimated working regions identified as the dashed squares in Figure 5 consider g in the range between 3.75 mm and 4.25 mm, and consider $\beta_{max}$ to fall between 120° to 140°. Considering these value ranges predicts $\theta_{max}$ ranges of between 104°-140° and 140°–182° for the $L^{'}$ of 30 mm and 40 mm designs respectively. The $\theta_{max}$ values in this case estimate maximum bending angle under the corrugation opening rather than the absolute maximum achievable with the additional consideration of appreciable strain in the OA channel wall; thus, the actual maximum achievable bending angles for the designs should be correspondingly higher. It is, however, the aim to design the OA such that the majority of the working range falls within the region dominated by corrugation opening; such to avoid the need for excessive pressure and non-linear bending responses.

**TABLE 1:** Geometric properties of the three-channel origami design.

Design Parameter	Definition	Chosen value for study
f	Wall thickness between central hollow channel and internal origami pattern	0.49 mm
j	Wall thickness between outer and inner origami features	1.59 mm
h	Thickness of the diagonal origami wall	0.92 6 mm
$C_{L}$	Corrugation wall length	1.6 mm
e	Pitch of corrugated pattern	2 mm
g	Radial distance	3.75 mm–4.25 mm
β	Angle between corrugated zig-zag wall pattern	77.36^o^
L	Overall length	41.90 mm and 51.90 mm
$L^{'}$	Corrugated length	30 mm for L = 41.90 mm 40 mm for L = 51.90 mm
N	Number of corrugated pattern (no unit)	20 for $L^{'}$ = 40 mm 15 for $L^{'}$ = 30 mm
D	Overall diameter	8.5 mm
d	Diameter at which inflatable channels were created	2.77 mm
b	Inner most channel for allowing endoscopic camera	1 mm

## Experimental Evaluation

As the intended endoscope use-case for the OA is via connection to manually controlled syringe pistons, representative evaluation was performed under volumetric control. In addition, to establish the usability of the design for robotically driven applications and offer more direct comparison to the presented model, evaluation of the OA was also performed under pressure controlled conditions.

**FIGURE 6:**
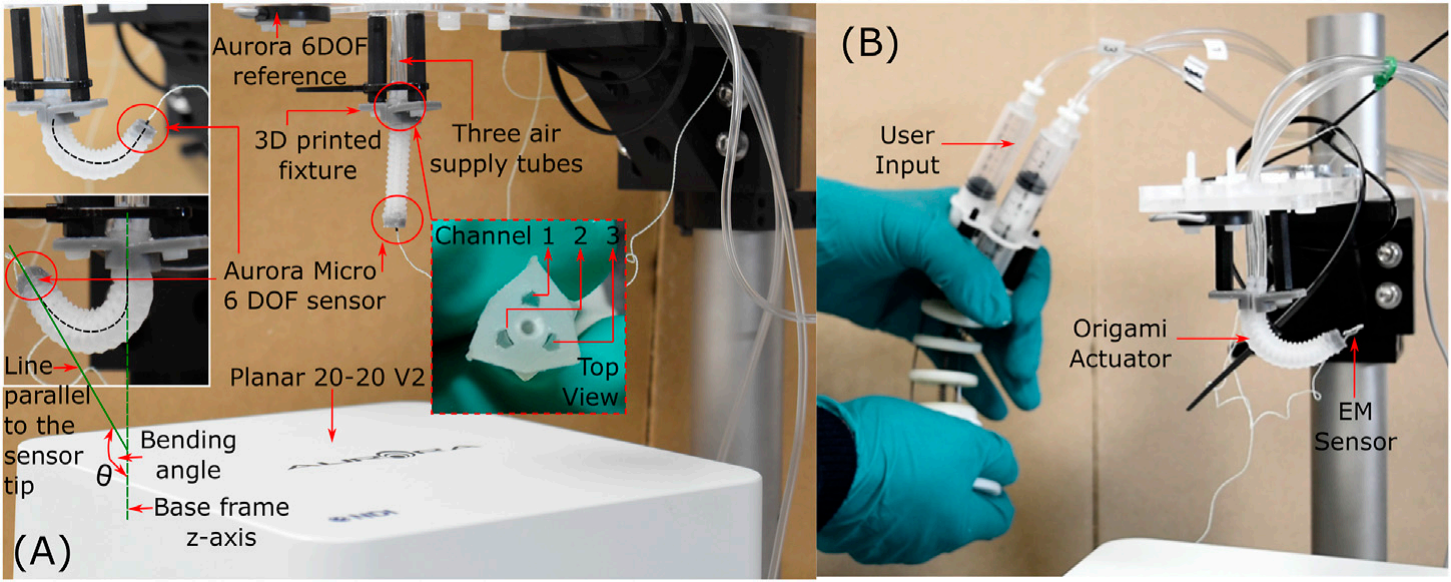
Experimental setup for testing **(A)** OA under volumetric and pressure conditions **(B)** OA after endoscopic integration.

The experimental setup (Figure 6A) for this evaluation comprised of a 3D printed (Grey Pro; Formlabs, United States) fixture which mounted the OA. The central hollow channel (sealed with closing cap in Figure 2) in the OA was utilized to fix an electromagnetic sensor (Aurora Micro 6DOF sensor tool, NDI, Canada). An electromagnetic sensing platform (Planar 20–20 V2) was used to measure the actuator tip position in reference to a fixed marker (Aurora 6DOF Reference, NDI, Canada) located at known proximity to the actuator base. The mounted end of the actuator was connected via three-way channel to three air supply tubes. Other components of this experimental setup comprised of, i) three 10 cc pneumatic syringes pressurized via controlled linear stages (actuated by a stepper motor) as detailed in (Chandler et al., 2020), ii) a pressure regulator (ITV0010-3BL, SMC Pneumatics, United States), and iii) three equal-length pneumatic lines connected to each channel of the actuator.

### Uniaxial Volumetric Characterization of Origami Actuator

Three pneumatic syringes pressurized using linear stages were used to inflate each channel (1, 2, and 3) individually in sequential steps of 0.069 ml to reach the maximum volume of 3.44 ml (refer to Table 2). This volume was chosen based on provisional testing with channel 3 of the OA with the insight of above criteria for reaching a bending angle within the theoretical range. The same volume of air was used to inflate channel 1 and 2. Following inflation, the channels were deflated to reach neutral position again in sequential steps of 0.069 ml. The bending angles and tip positions were measured throughout with reference to the static base position of the mounted OA (refer Figure 6A). As air is compressible, the influence of volume-rate was also explored with two feed rates (specifically at 0.5 and 1.5 ml.s^-1^); tested over the same volume range. The trial was repeated, five times under both the feed rate conditions. This test regime was applied to both variants of OA ($L^{'}$ = 30 mm and $L^{'}$ = 40 mm) with the test volumes for $L^{'}$ = 40 mm being increased due to its large internal volume, as identified in Table 2.

**TABLE 2:** Actuator testing parameters for pressure and volumetric control.

Actuator Length (mm)	Channel no	Peak pressure (kPa)	Angle at peak pressure (°)	Peak Volume (ml)	Angle at peak volume (°)
30	1	48	145	3.44	112
30	2	42	159	3.44	152
30	3	42	129	3.44	135
40	1	45	128	4.20	134
40	2	38	180	3.65	169
40	3	46	158	3.86	145

### Uniaxial Pressure Characterization of Origami Actuator

The pressure regulated testing on the actuator was performed using the same experimental setup detailed above (refer Figure 6A) with the controlled syringe being replaced with an electronically controlled pneumatic regulator (ITV0010-3BL, SMC Pneumatics, United States). This was connected sequentially to each channel-under-test and the pressure controlled using a bespoke software interface (LabVIEW, National Instruments, United States) from atmospheric up to a pre-determined peak pressure (as detailed in Table 2) and back down to 1 atm in stepwise fashion with 0.5 kPa steps per second. The trial was again repeated five times for the pressure regulated condition to a maximum of 150^o^ angular bending (for both variants of OA with $L^{'}$ = 30 mm and $L^{'}$ = 40 mm).

### Repeat Cyclic Testing

Suitable durability and repeatability are essential for effective use in the intended endoscopic application. The proposed OA is designed for single-use upper gastrointestinal screening and an ideal time span for such inspection is 15 to 20 min. An ideal number of cyclic bending (up to high angles) in such a scenario is estimated to range up to ∼50 cycles. Hence, cyclic testing was conducted to assess durability of the design and determine influence of repeated pressurized and volumetric actuation on bending performance of the OA. Using a pressure rate of 10 kPa steps per second and volumetric rate of 0.5 ml s^-1^, 50 repeated cycles were performed (using the experimental setup in Figure 6A) on channel 2 under the conditions identified in Table 2.

### Endoscopic Integration of Actuator

To evaluate the potential of the origami actuator design in an endoscopic application, each channel was connected to an independent syringe whose pistons are driven via a multi-backbone continuum joystick; as detailed in (Garbin et al., 2018b); as shown in Figure 6B. The user manipulated the joystick for 60 s through full range of motion to induce 3D articulation into the actuator, as required for endoscopic inspection. The tip pose of the actuator was simultaneously recorded.

### Varying Tip Load of Actuator

Since the OA is designed for upper gastrointestinal endoscopy, it may encounter possible tight orifices at the vocal folds, esophagus, and the duodenum. The OA was evaluated for deflection in the bending angle as a function of tip load. A mass, representing 0g (no-load), 0.5 g (estimated mass of the endoscopic camera, (Fujikura, 2021)) and 2 g (equivalent to OA mass) was placed at the tip of OA. Angular deflection of the actuator tip was recorded for the OA under pneumatic pressure and volume control for each tip load.

## Results

### Bending Behaviour for Volumetric and Pressure Control of Origami Actuator

**FIGURE 7:**
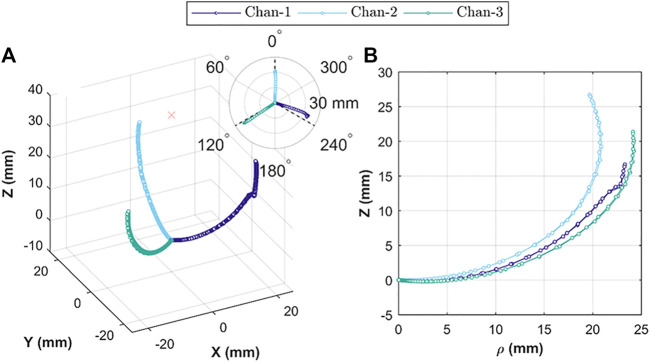
Actuator tip position during Independent chamber actuation; showing **(A)** relative 3D tip position for actuation under volumetric (*n* = 10) and pressure (*n* = 5) control; insert showing relative bending planes for the three chambers; and **(B)** tip position in cylindrical coordinates, comparing tip trajectory for each chamber under volumetric control (*n* = 5).

The 3D tip positions for each channel-test condition combination are shown in Figure 7A. This 3D plot is a combination of all repeats (total *n* = 15) for the $L^{'}$ = 30 mm OA tip for the two conditions of volumetric characterization (i.e., 5 repeats for 0.5 and 1.5 ml s^-1^ respectively) and pressure regulated characterization (5 repeats). It is apparent that each channel repeatedly follows a specific path regardless of volumetric or pressure control, and tends to move through a specific plane aligned with the global *z*-axis at ∼120° spacing. A comparison between the paths taken for each channel is shown in Figure 7B by overlapping the bending plane with azimuth φ set to 0°. The OA tip positions can be seen in cylindrical coordinates (for clarity only volumetric control at 0.5 ml s^-1^ shown, *n* = 5) of axial distance ρ and axial coordinate height Z. The paths show similar form with a scaling in radial distance (ρ direction) and varied maximal axial coordinate height values (Z direction).

**FIGURE 8:**
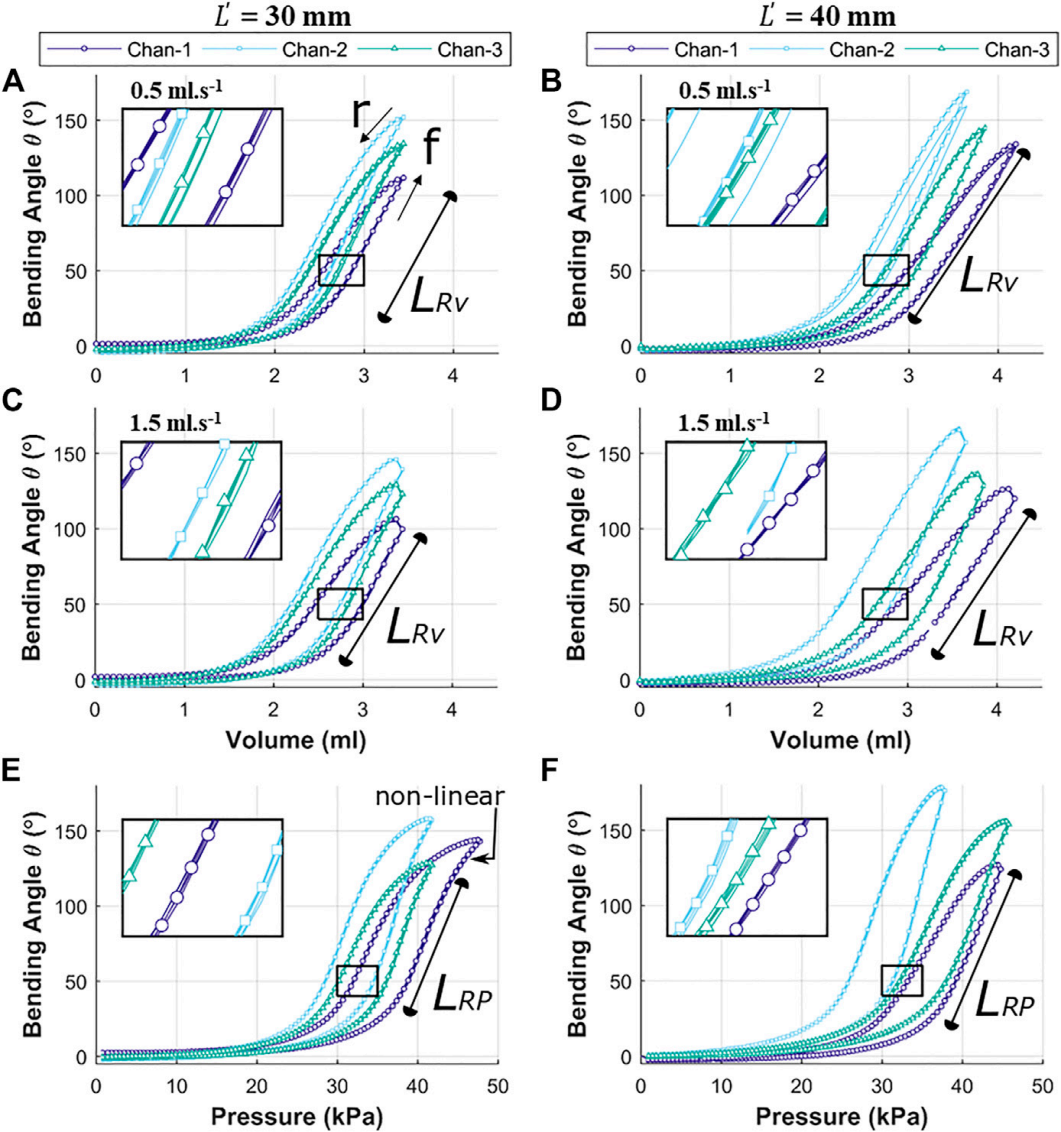
Independent chamber input to bending angle θ characterization for actuator designs of length L = 30 mm and L = 40 mm; under volumetric control at 0.5 ml s^-1^ **(A,C)** respectively, and volumetric control at 1.5 ml s^-1^ **(B,D)** respectively; pressure control at 0.5 kPa s^-1^ **(E,F)**. For each case, lines for 5 independent repeats are presented (as indicated in the inset callout views) with the median test also showing specific measurement points.

The reachability of the OA (specific position or bending angle) is dependent upon the rate of volumetric or pressure control and direction of the inflation and deflation cycle. Both the factors induce hysteresis in the actuation behavior. Figure 8 shows the hysteresis path for volumetric control (A to D) and pressure control (E to F) for both the variants of OA, i.e., with $L^{'}$ = 30 mm and $L^{'}$ = 40 mm.

As volume or pressure rise to inflate the OA (cycle, f), the bending behavior remains relatively flat initially (0 to 1.5 ml or 0 to 20 kPa) before it begins to deform (non-linearly between 1.5 to 2.5 ml or 20 to 35 kPa) significantly. This bending deformation is observed to be approximately linear between 2.5 to 3.3 ml (for $L^{'}$ = 30 mm), 2.5 ml–4.2 ml ($L^{'}$ = 40 mm), 35 to 42 kPa (for both $L^{'}$ = 30 mm) and 35 to 45 kPa (for $L^{'}$ = 40 mm). Considering the linear response ($L_{RV}$) of the bending angle with a change in volume at 0.5 mls^-1^ (Figures 8A,B), it can be understood that the increase in volume actuated the OA by unfolding the corrugation, with minimal contribution from the axial strain of the OA channel walls. As maximum volume is reached and the direction reversed (cycle, r), there is an altered return path due to stress relaxation in the material, which implies that less volume or pressure is required to hold in the same angular position. This resulting hysteresis behavior is exaggerated further for the 1.5 mls^-1^ volume rate tests (Figures 8C,D) as the stress relaxation and compressibility of air create a delayed response from the actuator. In these high volume-rate cases, a large linear response region $L_{RV}$ is again seen.

The linear response for pressure variation (caused by unfolding of the corrugation) in Figure 8E is observed to culminate with a non-linear region towards the maximum pressure, which implies appreciable elastic strain of the outer wall of the channel being induced to support further bending. The longer linear range ($L_{RP}$) of the bending response in Figure 8F means that increase in actuator length delays this non-linear response. The maximum linear bending angle $\theta_{max}$ ($L_{RV}$) across different channels was observed to be between 105^o^ to 150^o^ (for $L^{'}$ = 30 mm in Figures 8A,C) and no visible saturation was observed for angles up to between 120^o^ to 175^o^ (for $L^{'}$ = 40 mm in Figures 8B,D). Pressure control, again demonstrates hysteretic behavior of OA, with the hysteresis trajectory for longer OA (i.e. $L^{'}$ = 40 mm) showing less tapering at high bending. This demonstrated the possibility to reach higher bending angles in comparison to short OA (i.e. $L^{'}$ = 30 mm). The linear range ($L_{RP}$) of the maximum bending angle in Figure 8E varied from 110^o^ to 140^o^ (for 0.35 to 0.42 bar) and in Figure 8F varied from 130^o^ to 175^o^ (for 0.35 to 0.45 bar). This range of bending angle complies with the predicted range of 104°-140° and 140°–182° for the L' of 30 mm and 40 mm designs respectively, shown in Figure 5.

The experimental results show the air volume or pressure required to reach specific bending angles in the range of 0–150^o^. These values were observed to be varied for each inflatable channel, mainly due to non-linear behavior of elastomeric material, printing error/precision in mold fabrication and human error in the molding process of the OA. However, similarity in the shape of the hysteresis trajectory is shown in the bending behavior of each channel with high repeatability for each case (inset in all these graphs show an enlarged view the overlapped data points for repeated trails (*n* = 5)). This behavior may be numerically accounted for to determine necessary channel-specific input parameters to achieve the same desired angular bending.

### Change in Bending Behavior for Repeated Cyclic Loading

**FIGURE 9:**
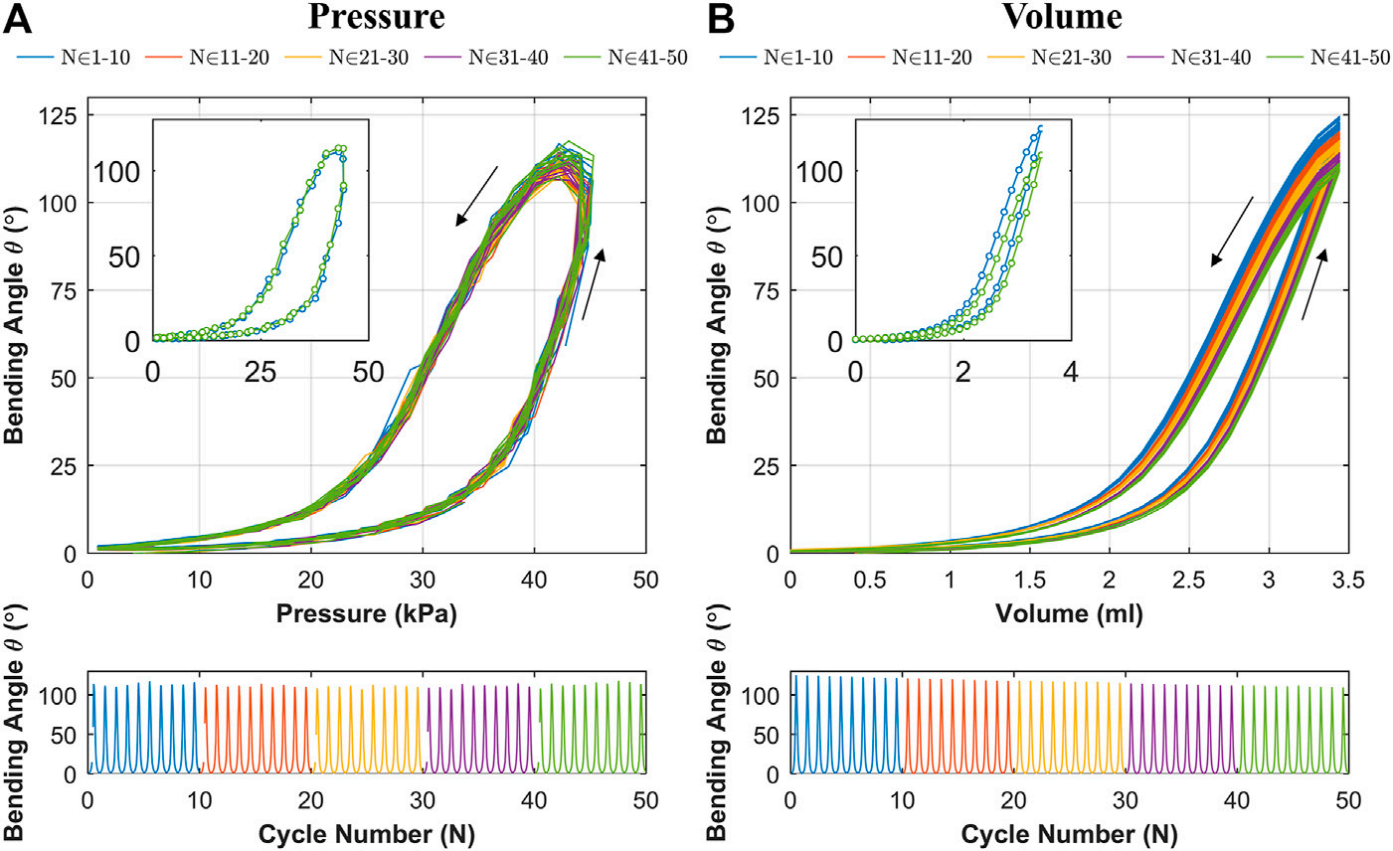
Bending angle (θ) vs **(A)** pressure at 10 kPa s^-1^ and **(B)** input volume at 0.5 ml s^-1^ over 50 test cycles (grouped in sets of 10 cycles) at 0.5 ml s^-1^; showing (upper) bending angle vs input; insert showing first (N=1) and last (N=50) cycles; and (lower) bending angle as a function of cycle number.

The results for cyclic testing under pressure and volumetric control are shown in Figures 9A,B respectively. It is evident from Figure 9A that the hysteresis loop remains consistent across cycles with no significant drop in the peak bending angle. This suggests no appreciable change in material characteristics through cyclic actuation. Under volumetric testing (Figure 9B), a moderate drop in the peak bending angle is noted as the cycles progress, which is highlighted by the separate first and last cycles shown. The drop in the peak bending angle between first and last cycle was 15.6^o^, representing a 12.5% reduction. As material changes are shown to be negligible through the cyclic pressure tests, the drop may be due to thermodynamic losses (resulting from cyclic compression/decompression of air leading to a loss of peak pressure) or any possible air loss from the actuator or connections. This shows that the OA is stable under cyclic loading from a material fatigue perspective (within the range tested), and thus represents a potentially suitable expected life for a single-use endoscope.

### Actuator Workspace With Endoscopic Integration

**FIGURE 10:**
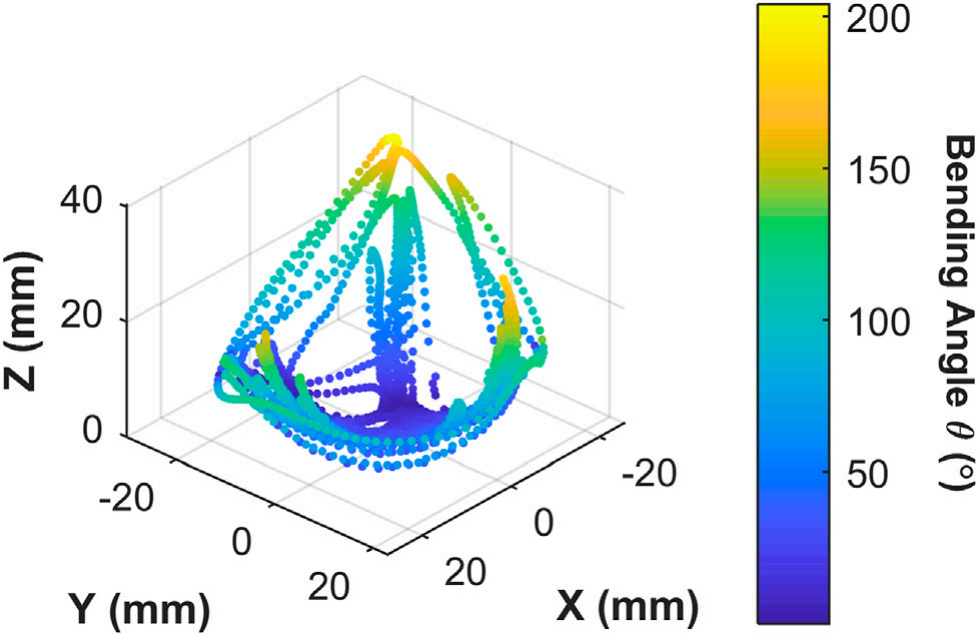
Origami actuator range of motion under manual user input control for 60 s; showing 3D positions covered by the actuator tip; color map represents the tip bending angle with respect to the z-axis.

The 3D trace in Figure 10 shows the actuator tip position (for $L^{'}$ = 30 mm) and its 3D workspace. As the user input was not constrained to the testing volumes presented in Table 2, the maximum bending angle was observed to be much higher; reaching consistently between 150–200° when aligned with a specific channel bending plane.

### Influence of Tip Loading on the Bending Angle

**FIGURE 11:**
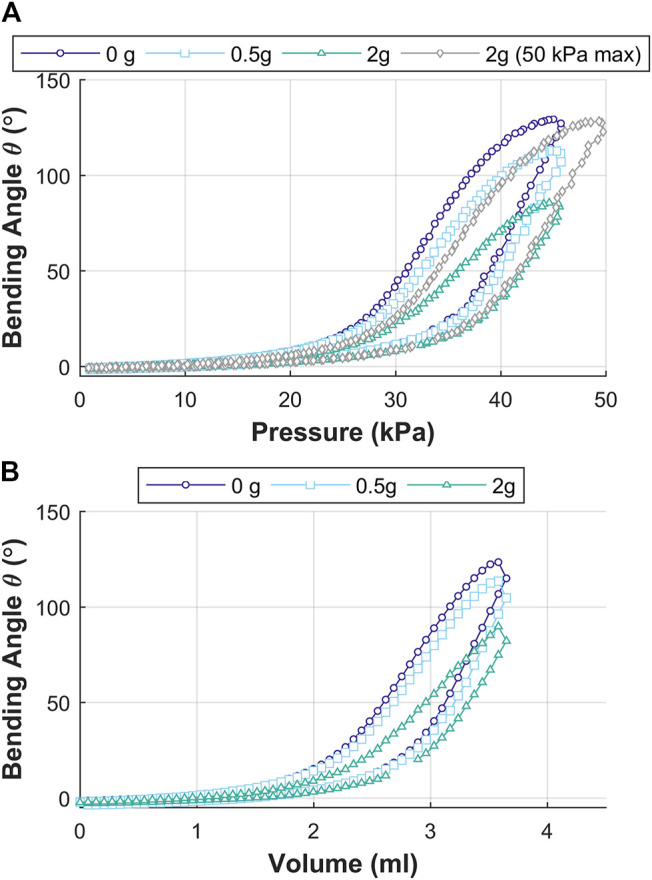
Influence of tip loading on the bending angle θ for actuator designs of length L = 30 mm under tip loads of 0 g (no load), 0.5 g (estimated camera mass), and 2 g (equivalent to OA mass); for: **(A)** pressure actuation showing influence of tip loads for fixed pressure and max load (2 g) at increased pressure to restore bending angle performance, and **(B)** volumetric control.

Figure 11 shows the results for influence of tip loading on the bending angle (θ) for the actuator designs of length L = 30 mm under no load (0 g), estimated camera mass load (0.5 g), and equivalent mass to the OA load (2 g). It is apparent that an increase in tip load results in reduced bending performance for the same applied pressure or volume. It is observed from Figure 11A that a rise in pressure allows a peak bending angle equivalent to the no-load condition. Thus, OA can counteract against the load introduced and restore the no-load bending angle performance with increased pressure actuation. Controlled bending performance can also be obtained by increasing the supplied air volume under volumetric control.

## Discussion

The above presented actuator design incorporates a unique origami pattern (i.e., PFP) in a monolithic, single material, three-channel structure of 8.5 mm diameter. The use of an origami pattern offers benefits like: i) production of corrugated profiles with the actuator walls, ii) flexibility in bending and allowance for continuous change in the bending plane, iii) introduction of three actuation and one endoscopic camera channel within minimal diameter, and iv) compact fabrication and production of actuator without damage in an elastomeric material (Dragon Skin 10). The choice of Dragon Skin 10 was a balance between stiffness for endoscopic applications and flexibility (bending performance) for a large range of motion at low pressure. Dragon Skin 10 offers a mid-range stiffness for soft robotic application (typically soft robots ranging from a 00–10 to >65A shore hardness). It is envisioned that this stiffness will be increased by the addition of a camera through the central lumen of the OA. Hence, the combination of both the material properties and inserted camera may offer the required stiffness for endoscopic applications.

Some of the design parameters of the OA (refer to Figure 3) are number of corrugated channels (n), total length (L), corrugated length (L'), outer diameter (D), diameter at which corrugation begins (d), and central lumen diameter (b). The OA could be scaled to different applications by varying these design parameters. Some examples of this optimization process can involve reducing the number of corrugated channels for adapting to smaller diameters (for e.g., nasal endoscopy or pediatric endoscopy). The length of the OA could be varied for adapting to larger diameter applications like colonoscopy etc. The central lumen diameter could be increased to adapt a biopsy forceps and additional channels for suction, insufflation etc. could be added.

The actuation of the OA shows controlled bending performance of the tip resulting from the opening of the corrugated features and elastic strain in the material of the outer wall of the channel. The OA was also tested for tip-load carrying capability through variation of pneumatic pressure and volume. An increase in tip load resulted in reduced bending performance if the same (no load) pressure or volume was applied. Further increase in pneumatic pressure helped in obtaining bending performance equivalent to no-load condition. This shows that the OA is stable under higher loads and able to counteract against the load introduced through a partly occluded duct or contact with an internal organ (during endoscopy).

Though the OA is made with a corrugated pattern, the OA is designed to be used for gastrointestinal endoscopy along with a lubricating gel to minimize friction during insertion. As mentioned earlier, soft robotic pneumatic actuators should be safe for the patient and the clinician with an ideal range of bending angles with repeatable performance between 100–200^o^. These aspects represent important considerations and will be further explored as part of future *ex vivo* tissue tests.

The theoretical maximum bending angle range (from corrugation opening, $\theta_{max}$ ) of 104°-140° and 140°–182° for the $L^{'}$ of 30 mm and 40 mm appear to comply with experimental results, overall $\theta_{max}$= 105^o^ and 175^o^ (in Figure 8). The OA shows a bending response with a segment of linear response with variation in volume $\left(L_{RV}\right)$ and pressure $\left(L_{RP}\right)$. The linear response lies in an estimated bending angle range of 30^o^ to 150^o^, which is useful for endoscopic application (Garbin et al., 2018b). It is important to note that the observed experimental bending angle range is for each channel of the OA, which means that a 3D workspace of the actuator will cover an area larger than the hemispherical surface (maximum approximate θ ≈ 300^o^). The observed range of bending angle is obtained for an actuation volume of 2.5 to 3.5 ml and pressure of 35 kPa–45 kPa (or 0.35–045 bar) (Figures 8E,F). A comparison of the actuator bending performance with various other existing soft robotic pneumatic actuators is represented in Table 3.

**TABLE 3:** Comparison of performance characteristics of elastomeric actuators.

Elastomeric Actuators	Maximum Pressure	Diameter (mm)	Inflatable channel Length (mm)	Maximum bending angle	Packing efficiency (Number of channels/actuator diameter)	Curvature (Max bending angle/Actuator length)
Lenssen et al. (2019)	0.97 bar	25	30	35^o^	0.12	1.17
Suzumori et al. (1997)	4 bar	16	48	90^o^	0.19	1.88
Elsayed et al. (2014)	0.1 bar	25	55	140^o^	0.12	2.55
Naghibi et al. (2019)	0.45 bar	28	40	100^o^	0.11	2.50
Chandler et al. (2020)	0.5 to 0.75 bar	9.6	50	181^o^ to 222^o^	0.31	3.62 to 4.44
Martinez et al. (2013)	0.3 bar	12	200	360^o^ (spirals into helix)	--	--
Origami Actuator	0.35 to 0.42 bar	8.5	30	110^o^ to 140^o^	0.35	3.67 to 4.67
	0.35 to 0.45 bar	8.5	40	130^o^ to 175^o^	0.35	3.25 to 4.375

It can be observed that various existing robotic pneumatic actuators have been able to achieve a similar range of bending angle for higher pressure. For example, Lenssen et al. 2019 reports pressure of 0.55 bar bending of an actuator (diameter 25 mm) with a bending range up to 110^o^ though this actuator required outer sheath for constraining radial expansion. Suzumori et al. (1997) reported actuation pressure of four bars for a bending angle of 80^o^ in an actuator of 16 mm in diameter. A bending angle up to 140^o^ for a pressure of 0.1 bar was reported (Elsayed et al., 2014) in a 25 mm diameter prototype, though this actuator relied on radial expansion. The parallel helix actuator (Chandler et al., 2020) showed a minimum and maximum bending angle of 181^o^ to 222^o^ for 0.5 to 0.75 bar (for different prototypes) for a 9.6 mm actuator diameter. The results obtained for OA show improved performance, with low pressure actuation in a sub-cm diametrical size (i.e., D = 8.5 mm).

The uniaxial bending performance for the three channels (under volumetric and pressurized control) repeated along the same plane (for repeated trials *n* = 5), which is expected behavior of monolithic structures. However, maximum reachability for each channel was different (Figure 7B), which is likely caused primarily by variability between the resultant geometry for each channel; something easily exacerbated with the small features of the OA and in longer designs (e.g., $L^{'}$ = 40 mm). Similar phenomena were observed from the hysteresis curve profiles indicated in Figure 8, which showed common but different bending characteristics for each channel. Differences in reachability for each channel of the actuator can be numerically aligned or compensated for via user control (as in the presented use case). The PFP pattern (in Figure 1B) showed the capability of complete elongation and collapse of the structure (longitudinally). This capability was partially lost due to incorporation of three inflatable channels in the design, though the results show that it did not affect the bending performance of the actuator; which is a primary requirement in endoscopic applications.

The length of the actuator was chosen to match the typical length for the bending section of the commercial endoscopes for upper gastro intestinal inspection (although bending angle performance is a more critical parameter for complete visualization). Prototyping an actuator for a longer length remains a challenge based on the current 3D printing technology available and used to produce the intricate mold cores. Specifically, fabricating actuators of sub-cm diameter and longer than 50 mm (approximately) is challenging as errors from non-true mold cores and alignment errors are amplified, reducing fabrication repeatability. The resultant OAs demonstrate suitable bending performance for the upper endoscope application within a length of either 30 or 40 mm. It is envisaged that smaller corrugated PFP inner cores can be created with advanced additive manufacturing or wax casting technique (Figure 2A), which would allow to scale down even smaller OA diameters.

The proposed OA is designed for single-use upper gastrointestinal screening and an ideal time span for such inspection is 15 to 20 min (up to ∼50 cycles). No significant drop in peak bending angle and a consistent hysteresis loop was observed in Figure 9A, which suggested no appreciable change in material characteristics through cyclic actuation. Though, a moderate drop in peak bending angle was noted under volumetric control (Figure 9B). This characteristic drop may be due to thermodynamic losses (resulting from cyclic compression/decompression of air leading to a loss of peak pressure) or any possible air loss from the actuator or connections. Though, this small reduction can be compensated manually by integrating the novel low-cost endoscope (Garbin et al., 2018a; Garbin et al., 2018b) or via momentarily resetting the system to atmospheric pressure at 0 piston displacement. In the case of the former, a maximum bending angle of 200^o^ was observed from Figure 10 along with the actuator profile during the shifting of the bending plane with no notable drop in performance over the 60 s test; suggesting suitability of the OA as a manually controlled endoscope.

## Conclusion

The OA design combines the structural benefit of PFP in a tubular construction, where the folds and creases of the corrugated pattern behave as pneumatically driven hinges avoiding the need for tendon wires or complex joints. This is the first time in soft robotics that origami-inspired corrugated design features are introduced and evaluated in a multi-channel, monolithic soft robotic actuator; which offers specific functionality for endoscopic applications. A repeatable fabrication technique has been presented for achieving this soft robotic actuator for endoscopic applications. A wide range of elastomeric and mold materials are compatible with the design principle, which may be coupled with variation in the corrugated pattern to meet specific OA requirements. Further research may focus on scaling the actuator diameter and length for making it suitable for nasal endoscopy purposes. The hollow channel introduced in the OA for an endoscopic camera can also be utilized for optical fiber integration, which may be useful for laser-based spectroscopy or other diagnostic and interventional techniques.

## Data Availability

The original contributions presented in the study are included in the article/Supplementary Material, further inquiries can be directed to the corresponding author.
